# Supplementary material for: Paleogene India-Eurasia collision constrained by observed plate rotation
Source: Nat Commun. 2023 Nov 10;14:7272. doi: 10.1038/s41467-023-42920-0 (PMC10638303; doi:10.1038/s41467-023-42920-0)
Supplement: Supplementary file 1 — Supplementary Information [file 41467_2023_42920_MOESM1_ESM.pdf]

Supplementary Information for

Paleogene India-Eurasia collision constrained by observed plate rotation

Xiaoyue Wu[1,2,3], Jiashun Hu[2*], Ling Chen[1,3], Liang Liu[4], Lijun Liu[1,5*]

1. State Key Laboratory of Lithospheric Evolution, Institute of Geology and Geophysics, Chinese Academy of Sciences, Beijing 100029, China.

2. Department of Earth and Space Sciences, Southern University of Science and Technology, Shenzhen 518055, China.

3. College of Earth and Planetary Sciences, University of Chinese Academy of Sciences, Beijing 100049, China.

4. State Key Laboratory of Isotope Geochemistry, Guangzhou Institute of Geochemistry, Chinese Academy of Sciences, Guangzhou 510640, China.

5. Department of Geology, University of Illinois at Urbana-Champaign, Champaign, IL 61820, USA.

*Correspondence to: * hujs@sustech.edu.cn; ljliu@illinois.edu*

This file includes:

Supplementary Figures 1 to 8

Supplementary Tables 1 to 4

Supplementary References

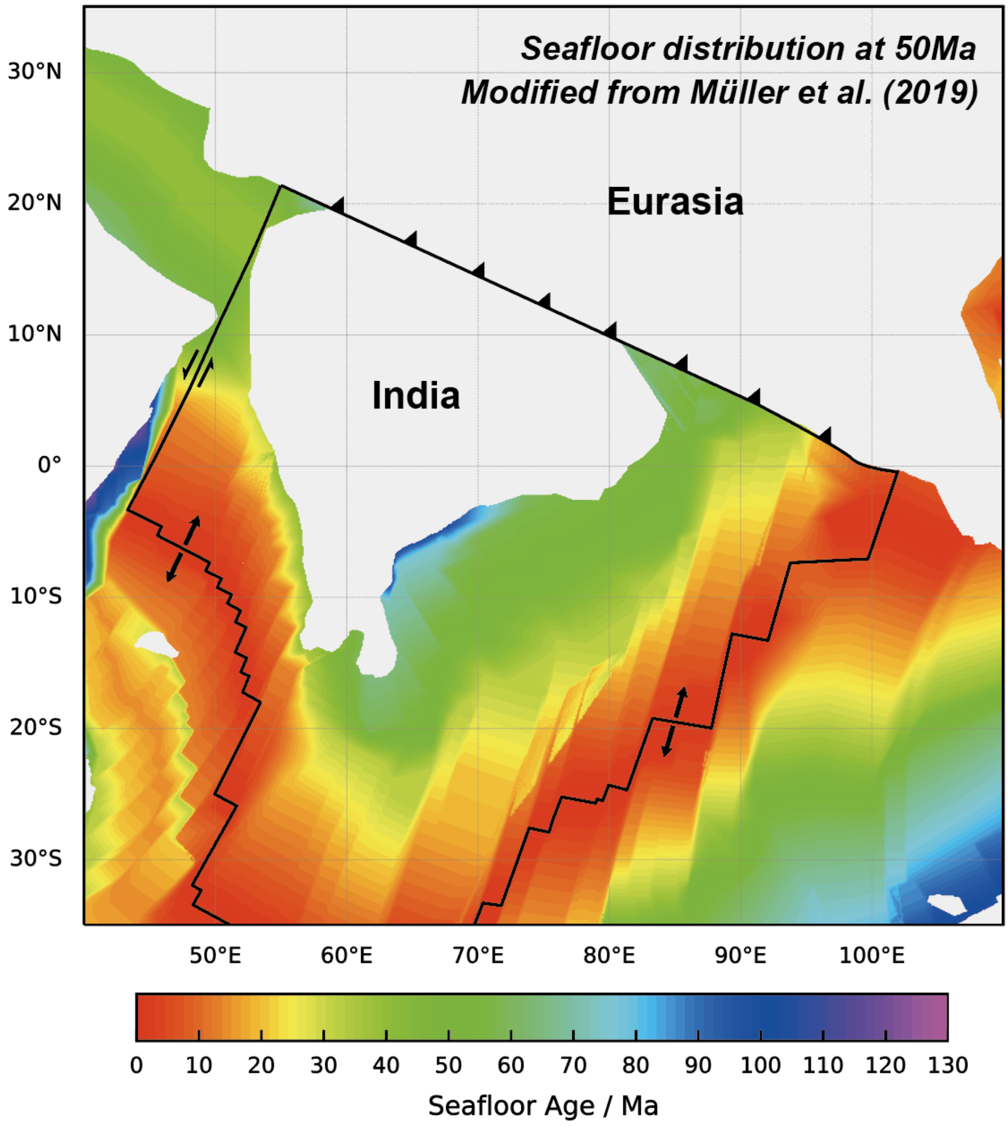

Supplementary Figure 1. Seafloor distribution at 50 Ma. The seafloor distribution is derived from ref.[1], with some simplifications around the collision zone so as to have a linear collision front. The oceanic lithosphere on the eastern side of the Indian continent is much wider than that on the west.

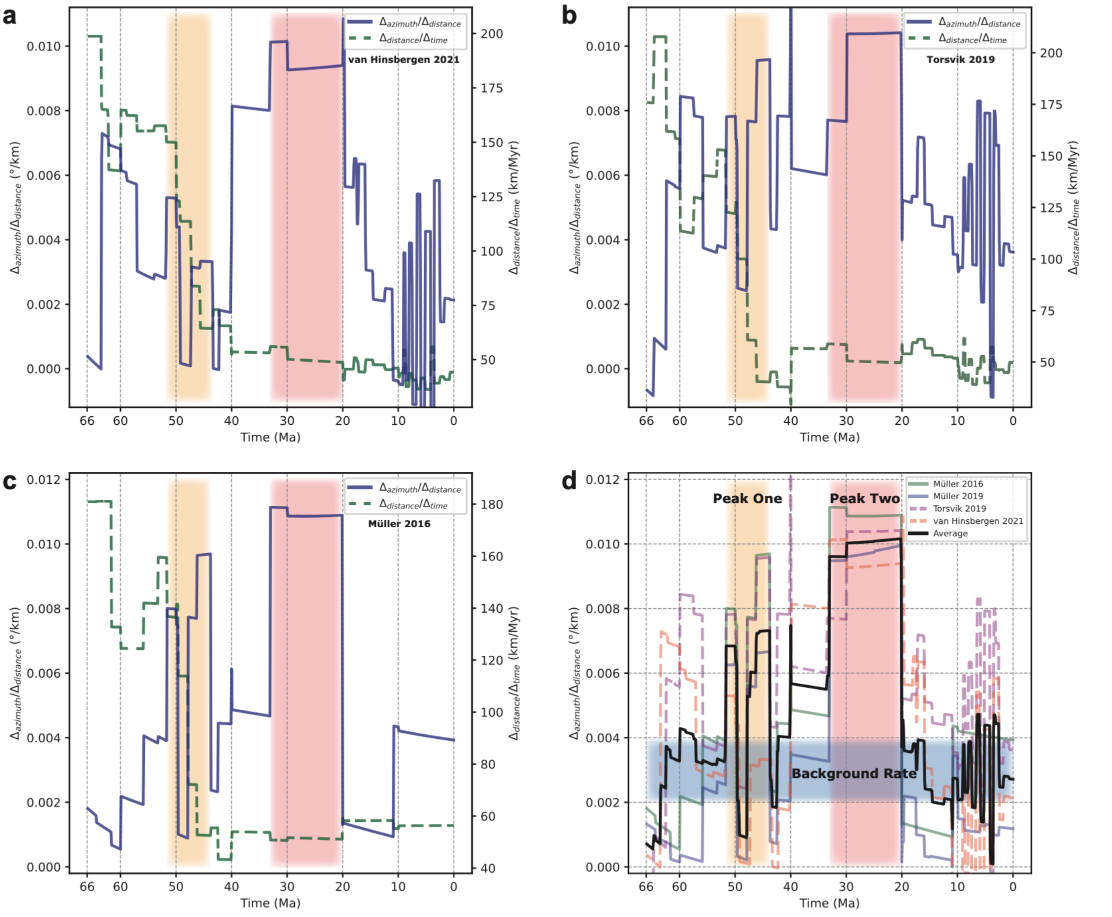

Supplementary Figure 2. Rotation rates and along-track velocity of the Indian plate based on other reconstructions. a, Rates based on ref.[2]. **b,** Rates based on ref.[3]. **c,** Rates based on ref.[4]. **d,** Compilation and the average rates. Details of these reconstructions are listed in Supplementary Table 1.

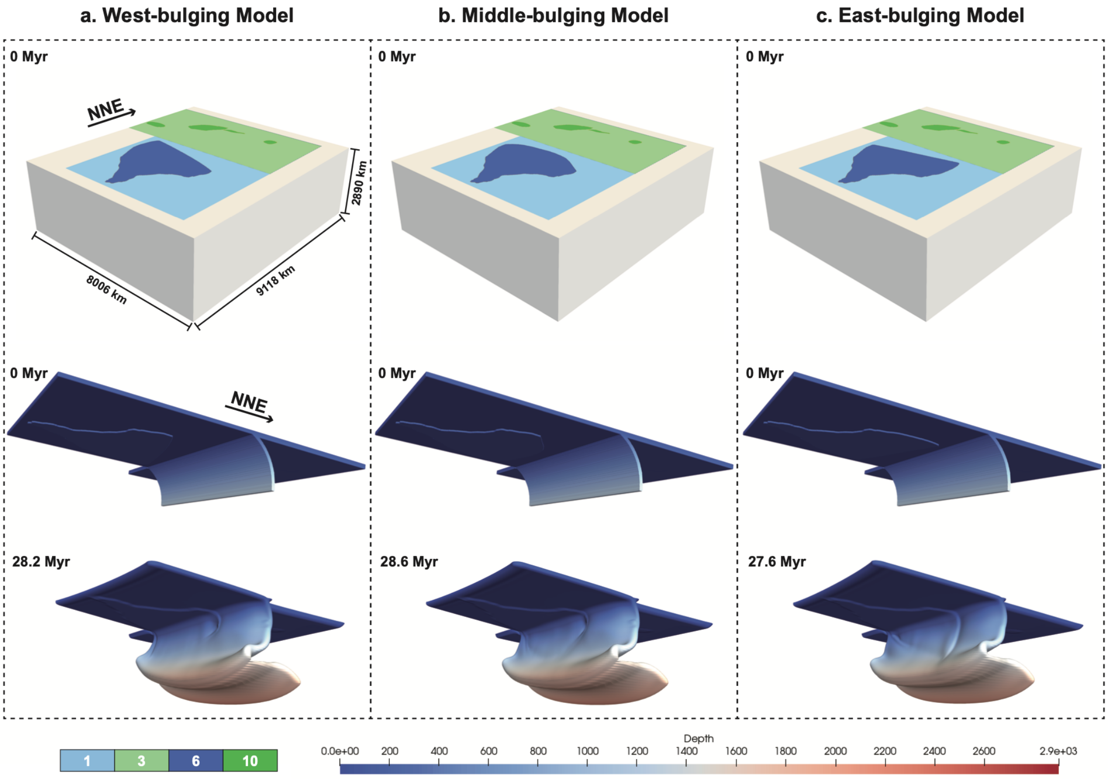

Supplementary Figure 3. Initial plate configuration and slab structures of the numerical models. a, west-bulging model. **b,** middle-bulging model. **c,** east-bulging model. The box on the top is the whole model domain shown in the Cartesian coordinate system. Different colors correspond to different compositions: 1-oceanic crust, 3-Eurasian non-cratonic continental upper crust, 6-Indian continental upper crust, 10-Eurasian cratonic upper crust. The plate structures in the middle and bottom rows are at their initial and final states. The color of the slab indicates depth, whose unit is kilometer (km).

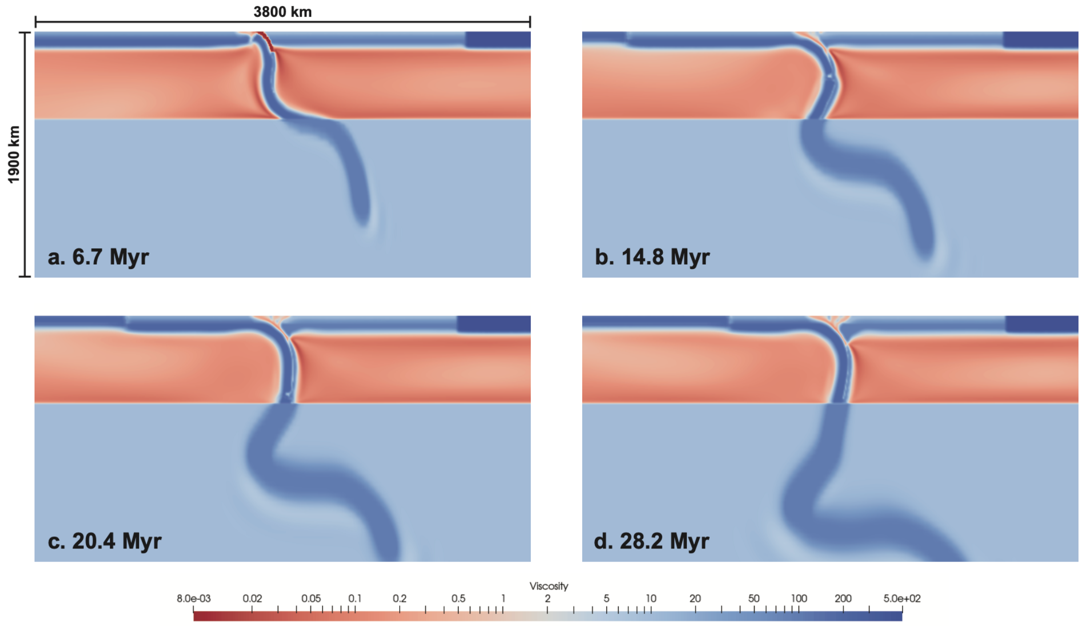

Supplementary Figure 4. The evolution of model's viscosity field. These profiles show the evolution of the viscosity field cutting through the bulge of west-bulging model at (**a**) the initial collision, (**b**) the first rotation rate peak, (**c**) the complete collision, and (**d**) the second rotation rate peak. The reference viscosity is 10^{21} Pa·s. The contact area between the subducting plate and the overriding plate increases with time after continental collision (**b**-**d**), implying a stronger coupling between the plates. The pre-existing oceanic slab buckles when penetrating the high-viscosity lower mantle.

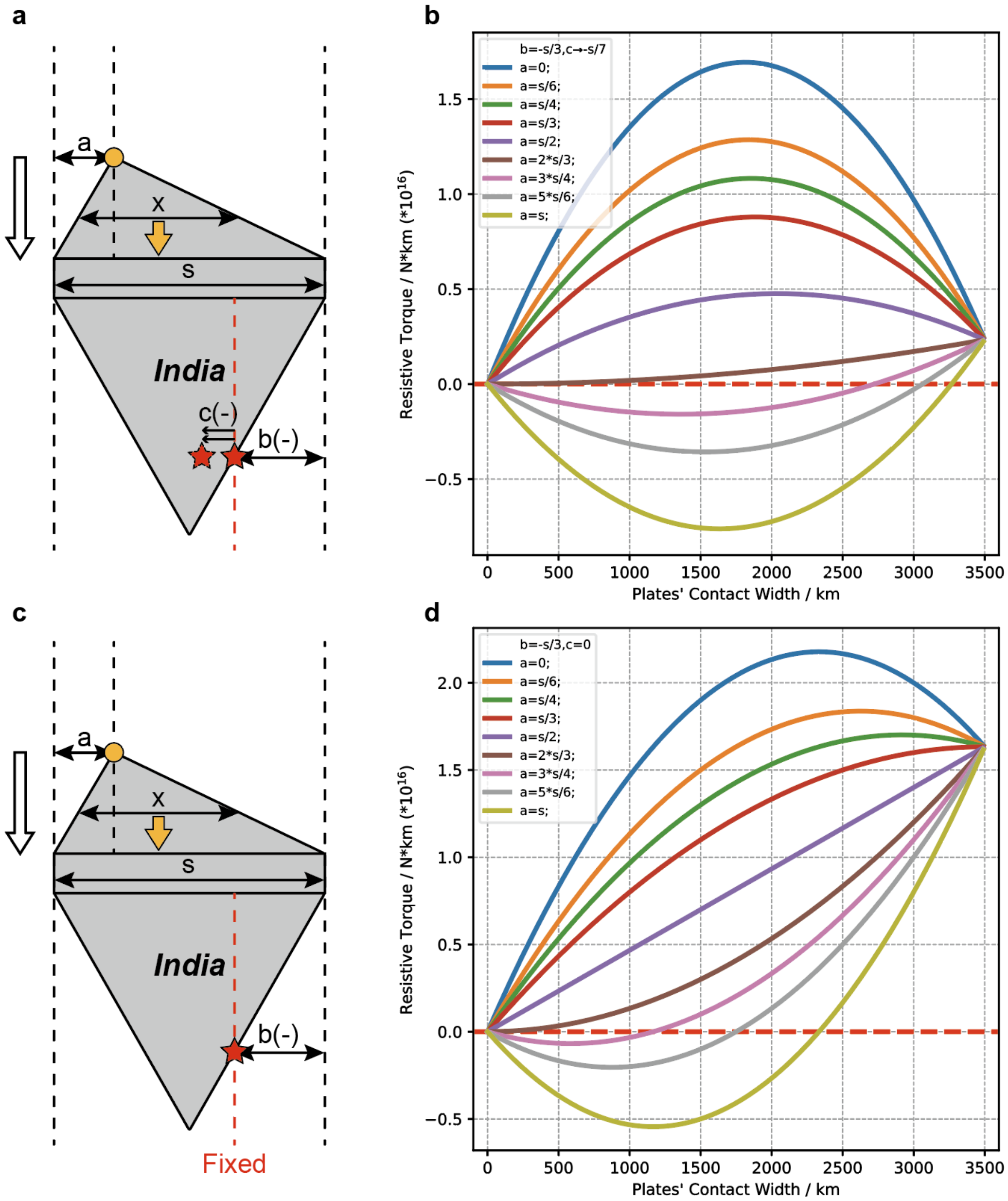

Supplementary Figure 5. A straightforward calculation of resistive torques. a, Diagram of the Indian continent before collision. The orange circle and the red star represent the bulge of Greater India and the center of mass of the Indian plate, respectively. The orange arrow represents the resultant resistive force. **b,** Calculated resistive torque versus contact width between the Indian and Eurasian continents. **c** & **d**, diagram and calculations when the offset of the mass center is not considered. We suggest that the west- and middle-bulging models are more comparable with the torque analysis considering the movement of the center of mass (**a**, **b**), while the east-bulging model is more comparable with the torque analysis without the movement of the center of mass (**c**, **d**). See Methods for detailed discussions.

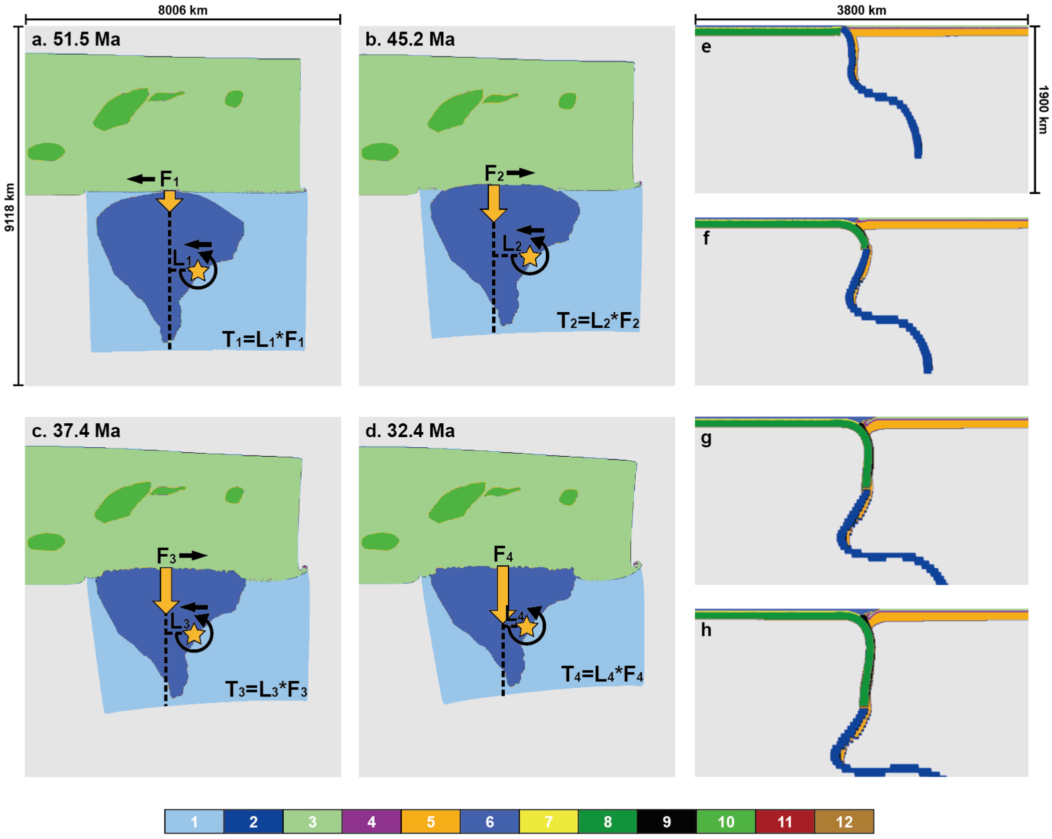

Supplementary Figure 6. Snapshots of the middle-bulging model at key stages. Map view at (**a**) the initial collision, (**b**) the first rotation rate peak, (**c**) the complete collision, and (**d**) the second rotation rate peak. (**e**)-(**h**) show the corresponding zoomed vertical cross-sections cutting through the bulge. Other captions are the same as in Fig. 2.

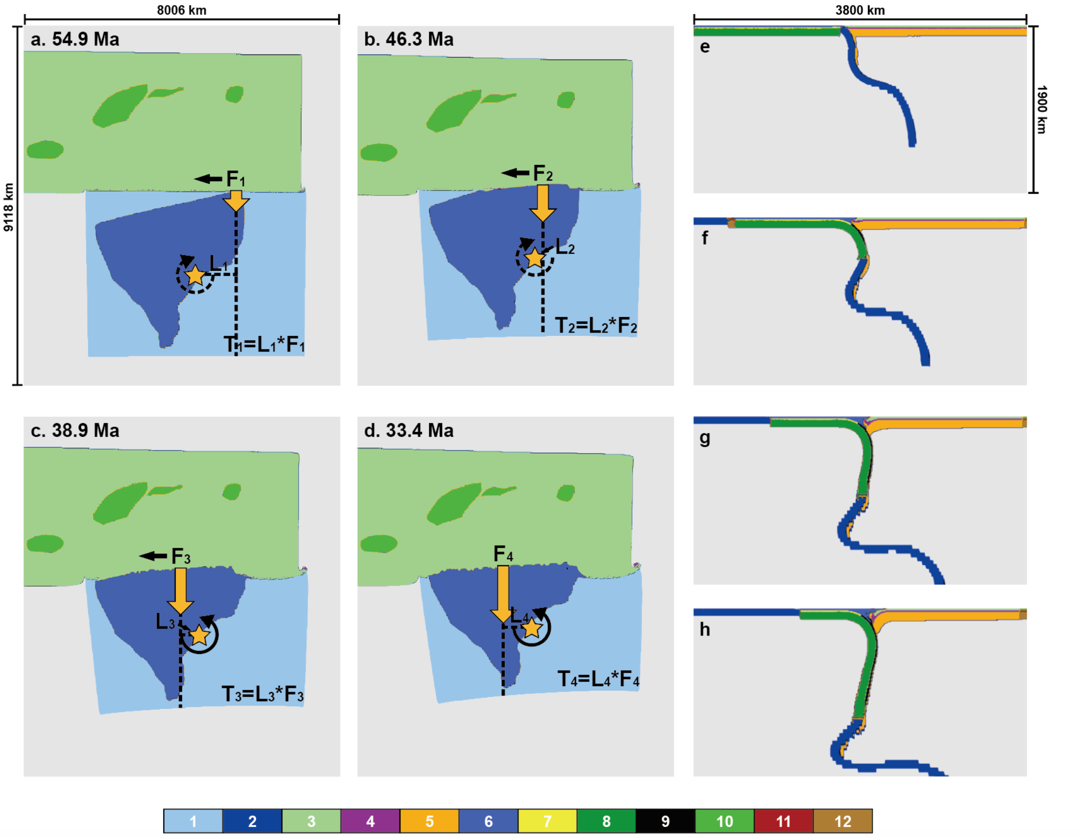

Supplementary Figure 7. Snapshots of the east-bulging model at key stages. Map view at (**a**) the initial collision, (**b**) the Stage Two in Fig. 3a, (**c**) the complete collision, and (**d**) the rotation rate peak. (**e**)-(**h**) show the corresponding zoomed vertical cross-sections cutting through the bulge. Other captions are the same as in Fig. 2.

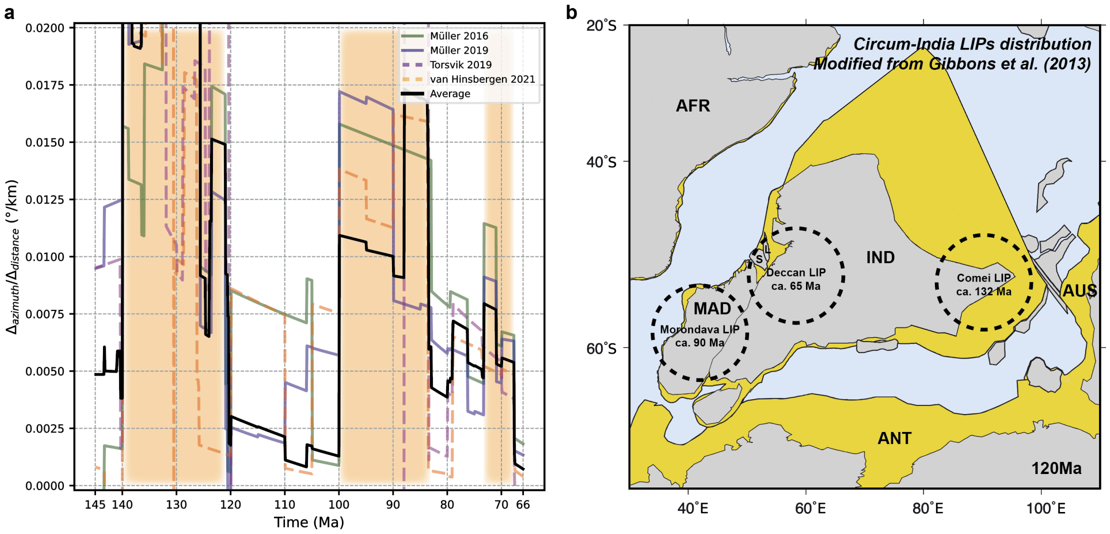

Supplementary Figure 8. Cretaceous rotation motion of the Indian plate and related large igneous provinces (LIPs). a. Rotation rate during 145-66 Ma, with three rotation peaks highlighted by shading. The rates and their average are calculated based on the plate reconstructions of refs.[1-4]. Methods and reference points are the same as those applied for the Cenozoic era. **b.** Distribution of circum-India LIPs erupted since the early Cretaceous. The Comei LIP and its associated plume were situated at the northeastern margin of the Indian plate[5]. The Morondava LIP, the Deccan LIP and their associated plumes were situated at the southwestern margin of the Indian plate[6]. The base map is modified from the 120 Ma reconstruction of ref.[7]. AFR: Africa, ANT: Antarctica, AUS: Australia, IND: India, MAD: Madagascar, L: Laxmi Ridge, S: Seychelles.

Supplementary Table 1. Details of the four Indian motion reconstructions during ~ 66-20 Ma.

Reconstructions	Relative Plate Circuits	Absolute Reference Frames
Müller 2019 (ref.[1])	India-Capricorn-(Antarctica)-Africa	Optimized mantle frame From ref.[8]
Müller 2016 (ref.[4])	India-Capricorn-(Antarctica)-Africa	Global moving hotspot mantle frame From ref.[9]
Torsvik 2019 (ref.[3])	India-(Capricorn)-Somalia-Africa	Global moving hotspot mantle frame From ref.[10]
van Hinsbergen 2021 (ref.[2])	India-(Capricorn)-Somalia-Africa	Slab-fitted mantle frame From ref.[11]

Supplementary Table 2. Vertical depth-dependent rheology parameters.

Parameters	Lithosphere	Asthenosphere	Transition Zone	Lower Mantle
Range(km)	0-64	64-410	410-660	660-2890
A_{dif}(Pa·s)	1.0×10^{20}	2.0×10^{21}	2.0×10^{21}	1.0×10^{22}
A_{dis}(Pa·s$^{1/n}$)	3.9×10^{8}	7.7×10^{9}	7.7×10^{9}	Not-Exist
E_{a}(kJ/mol)(dif)	135	135	135	162
E_{a}(kJ/mol)(dis)	470	470	470	Not-Exist
T_{off} (K)	130	130	130	130

Supplementary Table 3. Lateral composition-dependent rheology parameters.

Parameters	Oceanic Crust	Oceanic Lithospheric Mantle (LM)	Indian Lithosphere	Asian Non-Cratonic Lithosphere	Asian Cratonic Lithosphere
η_{c}	0.008	1	200 (Crust) 2000 (LM)	100 (Crust) 1000 (LM)	500 (Crust) 5000 (LM)
C_0(MPa)	50	500	100	100	500
C_{f}(Mpa)	10	100	20	20	100
μ_0	0.1	0.6	0.2	0.2	0.6
ε_{f}	0.1	0.2	0.2	0.2	0.2
$\eta_{\mathrm{cut-off}}$(Pa·s)	8×10^{18}-2×10^{23}	8×10^{18}-2×10^{23}	8×10^{18}-2×10^{23}	8×10^{18}-1×10^{23}	8×10^{18}-5×10^{23}

The $\eta_{\mathrm{cut-off}}$ denotes the upper and lower limits of viscosity considered in the models.

Supplementary Table 4. Plate geometric and physical parameters.

Parameters	Continental Upper Crust	Continental Lower Crust	Continental Lithospheric Mantle
Thickness (km)	20	15	85
Density (kg/m^3)	2700	3000	3340
Parameters	Oceanic Crust	Eclogitic Crust (Phase Change)	Oceanic Lithospheric Mantle
Thickness (km)	8	Depth > 80	72
Density (kg/m^3)	3000	3500	3340

Supplementary References

1. Müller, R. D. et al. A global plate model including lithospheric deformation along major rifts and orogens since the Triassic. *Tectonics* **38**, 1884-1907 (2019).
2. van Hinsbergen, D. J. et al. A record of plume-induced plate rotation triggering subduction initiation. *Nat. Geosci.* **14**, 626-630 (2021).
3. Torsvik, T. H. et al. Pacific-Panthalassic reconstructions: Overview, errata and the way forward. *Geochem. Geophys. Geosyst.* **20**, 3659-3689 (2019).
4. Müller, R. D. et al. Ocean basin evolution and global-scale plate reorganization events since Pangea breakup. *Annu. Rev. Earth Planet. Sci.* **44**, 107-138 (2016).
5. Zhu, D.-C. et al. The 132 Ma Comei-Bunbury large igneous province: Remnants identified in present-day southeastern Tibet and southwestern Australia. *Geology* **37**, 583-586 (2009).
6. Jiang, Q., Jourdan, F., Olierook, H. K. & Merle, R. E. An appraisal of the ages of Phanerozoic large igneous provinces. *Earth Sci. Rev.* **237**, 104314 (2023).
7. Gibbons, A. D., Whittaker, J. M. & Müller, R. D. The breakup of East Gondwana: Assimilating constraints from Cretaceous ocean basins around India into a best-fit tectonic model. *J. Geophys. Res. Solid Earth* **118**, 808-822 (2013).
8. Tetley, M. G., Williams, S. E., Gurnis, M., Flament, N. & Müller, R. D. Constraining absolute plate motions since the Triassic. *J. Geophys. Res. Solid Earth* **124**, 7231-7258 (2019).
9. Torsvik, T. H., Müller, R. D., Van der Voo, R., Steinberger, B. & Gaina, C. Global plate motion frames: toward a unified model. *Rev. Geophys.* **46**, RG3004 (2008).
10. Doubrovine, P. V., Steinberger, B. & Torsvik, T. H. Absolute plate motions in a reference frame defined by moving hot spots in the Pacific, Atlantic, and Indian oceans. *J. Geophys. Res.* **117**, B09101 (2012).
11. van der Meer, D. G., Spakman, W., van Hinsbergen, D. J., Amaru, M. L. & Torsvik, T. H. Towards absolute plate motions constrained by lower-mantle slab remnants. *Nat. Geosci.* **3**, 36-40 (2010).
